# GPER-mediated proliferation and estradiol production in breast cancer-associated fibroblasts

**DOI:** 10.1530/ERC-13-0237

**Published:** 2014-04

**Authors:** Haojun Luo, Guanglun Yang, Tenghua Yu, Shujuan Luo, Chengyi Wu, Yan Sun, Manran Liu, Gang Tu

**Affiliations:** 1 Department of Breast and Thyroid Surgerythe Second Affiliated Hospital of Chongqing Medical UniversityChongqing, 400010China; 1 Department of Endocrine and Breast Surgerythe First Affiliated Hospital of Chongqing Medical UniversityNo. 1 You-Yi Road, Yu-zhong District, Chongqing, 400010China; 2 Department of Gynecology and ObstetricsChongqing Health Center for Women and ChildrenChongqing, 400010China; 3 Key Laboratory of Laboratory Medical Diagnostics, Chinese Ministry of EducationChongqing Medical UniversityNo. 1 Yi-Xue-Yuan Road, Yu-zhong District, Chongqing, 400016China

**Keywords:** CAF, GPER, tamoxifen resistance, breast cancer

## Abstract

Cancer-associated fibroblasts (CAFs) are crucial co-mediators of breast cancer progression. Estrogen is the predominant driving force in the cyclic regulation of the mammary extracellular matrix, thus potentially affecting the tumor-associated stroma. Recently, a third estrogen receptor, estrogen (G-protein-coupled) receptor (GPER), has been reported to be expressed in breast CAFs. In this study, GPER was detected by immunohistochemical analysis in stromal fibroblasts of 41.8% (59/141) of the primary breast cancer samples. GPER expression in CAFs isolated from primary breast cancer tissues was confirmed by immunostaining and RT-PCR analyses. Tamoxifen (TAM) in addition to 17β-estradiol (E_2_) and the GPER agonist G1 activated GPER, resulting in transient increases in cell index, intracellular calcium, and ERK1/2 phosphorylation. Furthermore, TAM, E_2_, and G1 promoted CAF proliferation and cell-cycle progression, both of which were blocked by *GPER* interference, the selective GPER antagonist G15, the epidermal growth factor receptor (EGFR) inhibitor AG1478, and the ERK1/2 inhibitor U0126. Importantly, TAM as well as G1 increased E_2_ production in breast CAFs via GPER/EGFR/ERK signaling when the substrate of E_2_, testosterone, was added to the medium. GPER-induced aromatase upregulation was probably responsible for this phenomenon, as TAM- and G1-induced *CYP19A1* gene expression was reduced by *GPER* knockdown and G15, AG1478, and U0126 administration. Accordingly, GPER-mediated CAF-dependent estrogenic effects on the tumor-associated stroma are conceivable, and CAF is likely to contribute to breast cancer progression, especially TAM resistance, via a positive feedback loop involving GPER/EGFR/ERK signaling and E_2_ production.

## Introduction

The local microenvironment plays an important and intricate role in the progression of breast cancer. Increasing evidence suggests that activated stroma is a prerequisite for tumor formation (Barcellos-Hoff & Ravani 2000), the progression of ductal carcinoma *in situ* to the invasive stage (Hu *et al*. 2008), and the metastatic process (Karnoub *et al*. 2007). However, stromal cells have been revealed to inhibit the early stages of tumor progression, but to promote progression at the later stages (Proia & Kuperwasser 2005, Cirri & Chiarugi 2012). Importantly, the tumor:stroma ratio and the stroma type in primary breast cancer have been reported to be associated with patient survival including recurrence, distant metastasis, and death (de Kruijf *et al*. 2011, Ahn *et al*. 2012).

The cancer stroma is composed of diverse cell types including endothelial cells, immune cells, and fibroblasts, which are the most abundant cell type in breast cancer (Xing *et al*. 2010, Cirri & Chiarugi 2012). Considering that ‘tumors are wounds that do not heal’, ∼80% of fibroblasts in breast cancer stroma share some similarities, such as α-smooth muscle actin (SMA) expression, with myofibroblasts being activated during wound healing (Sappino *et al*. 1988). These fibroblasts acquire an aggressive phenotype and contribute to tumor growth, angiogenesis, invasion, and metastasis directly or indirectly by paracrine action of various growth factors, cytokines, proteases, and hormones including estrogens (Karnoub *et al*. 2007, Hu *et al*. 2008, Yamaguchi & Hayashi 2009, Xing *et al*. 2010, Cirri & Chiarugi 2012). Accordingly, these activated fibroblasts in the tumor stroma are designated cancer-associated fibroblasts (CAFs) and are recognized as co-mediators of tumor progression rather than as merely bystanders.

Estrogen is well recognized as a mitogen for breast cancer cells. Traditionally, estrogenic effects have been ascribed to the nuclear estrogen receptors (ERα and ERβ) that function as transcription factors binding to the regulatory response elements in the promoters of target genes (Pietras *et al*. 2005). However, estrogen also triggers rapid cellular events that are independent of transcriptional activity (Pietras & Szego 1975, Filardo *et al*. 2000, Pietras *et al*. 2005, Revankar *et al*. 2005). At present, the third ER, estrogen (G-protein-coupled) receptor (GPER) (also referred as GPR30), is largely accepted as the mediator of these ‘rapid’, ‘nongenomic’ effects (Prossnitz & Maggiolini 2009, Prossnitz & Barton 2011). GPER was identified in the late 1990s as a seven-transmembrane G-protein-coupled receptor (GPCR), belonging to a superfamily of membrane receptors traditionally recognized to mediate rapid signaling by modulating second messengers and kinase pathways (Owman *et al*. 1996, Carmeci *et al*. 1997, O'Dowd *et al*. 1998). In 2000, the 17β-estradiol (E_2_)-triggered rapid activation of ERK1/2 in breast cancer cells was found to correlate with GPER (GPER1) expression by Filardo *et al*. (2000). In 2002, the same group reported that GPER mediated the transactivation of the epidermal growth factor receptor (EGFR) to the MAPK signaling axis in response to E_2_ (Filardo 2002). In addition, GPER was reported to be associated with the modulation of calcium (Ca^2+^), cAMP, and phosphatidylinositol 3-kinase (PI3K) in the subsequent years (Filardo *et al*. 2002, Pietras *et al*. 2005, Revankar *et al*. 2005). Although the transcriptional effect of estrogen is almost exclusively correlated with ERα, GPER-triggered rapid signaling events have also been observed to regulate gene expression. Multiple genes, such as *FOS*, *BCL2*, cyclin D1, connective tissue growth factor (*CTGF*), and early growth response 1 (*EGR1*), have been included in the GPER-targeted gene list (Prossnitz & Maggiolini 2009, Madeo & Maggiolini 2010, Vivacqua *et al*. 2012). Accordingly, GPER has been shown to be involved in the proliferation, migration, chemoresistance, and metastasis of breast cancer (Wang *et al*. 2010).

Estrogen plays a critical role in the development and cyclic regulation of the mammary gland, which is composed not only of epithelium but also of stroma (Arendt *et al*. 2010). This raises the logical question of whether estrogen affects breast-cancer-associated stroma. Intriguingly, accumulating evidence related to GPER expression and its proliferative role in breast CAFs has been reported (Madeo & Maggiolini 2010, Pupo *et al*. 2012, Vivacqua *et al*. 2012). Madeo & Maggiolini (2010) revealed that GPER is exclusively expressed as an ER in mammary CAFs and induces the expression of C-FOS, cyclin D1, and CTGF in response to E_2_, confirmed at both the mRNA and the protein level, resulting in the promotion of proliferation. GPER has also been shown to be involved in the growth and migration stimulated by E_2_, 4-hydroxytamoxifen, and bisphenol A in breast CAFs (Pupo *et al*. 2012, Vivacqua *et al*. 2012). Thus, it can be proposed that estrogen, as an important factor in the breast cancer local microenvironment, is involved in cancer progression in a GPER-mediated CAF-dependent model.

Interestingly, tamoxifen (TAM), a selective ER modulator (SERM) targeting ERα, and its metabolite 4-hydroxytamoxifen have been recognized as agonists of GPER (Prossnitz & Barton 2011, Vivacqua *et al*. 2012), indicating a potential role of GPER in breast cancer TAM resistance. In clinical specimens, the co-expression of ERα (ESR1) and GPER has been found in ∼40% of the primary breast cancer cases and GPER expression, as an independent unfavorable factor, has been found to correlate with relapse-free survival in patients treated with TAM (Filardo *et al*. 2006, Ignatov *et al*. 2011). *In vitro*, TAM has been reported to enhance proliferation via sensitivity-enhanced GPER/EGFR/MAPK signaling in TAM-resistant MCF-7 cells, and knockdown of *GPR30* in MCF-7 cells has been shown to decrease the proliferation of cells exposed to TAM (Ignatov *et al*. 2010, Peralta *et al*. 2010). Furthermore, a TAM analog has been demonstrated to promote endometrial cell proliferation and aromatase gene expression, implying that GPER plays a positive role in estrogen production (Lin *et al*. 2009).

In the present study, we demonstrated that GPER is expressed in the stromal fibroblasts of primary breast cancer tissues and CAFs isolated from tumor tissues. E_2_, the GPER agonist G1 (1-(4-(6-bromobenzo[1,3]dioxol-5-yl)-3a,4,5,9b-tetrahydro-3H-cyclopenta[c]quinolin-8-yl)-ethanone, 1-[(3aS,4R,9bR-rel)-4-(6-bromo-1,3-benzodioxol-5-yl)-3a,4,5,9b-tetrahydro-3H-cyclopenta[c]quinolin-8-yl]-ethanone), and TAM activated GPER in breast CAFs and promoted proliferation and cell-cycle progression via the GPER/EGFR/ERK axis. Moreover, TAM and G1 induced *CYP19A1* gene expression and increased E_2_ production, also via the GPER/EGFR/ERK pathway, providing novel insights into the estrogenic effects on the breast cancer microenvironment and the induction of TAM resistance in a CAF-dependent manner.

## Subjects and methods

### Chemicals

E_2_, TAM, the GPER agonist G1, and the GPER antagonist G15 (4-(6-bromo-benzo[1,3]dioxol-5-yl)-3a,4,5,9b-tetrahydro-3H-cyclopenta[c]quinoline) were purchased from Sigma–Aldrich. The EGFR inhibitor AG1478 (AG), the ERK1/2 inhibitor U0126, and the PI3K inhibitor Wortmannin (WM) were purchased from Millipore (Temecula, CA, USA). All chemicals were solubilized in DMSO.

### Clinical specimens

A total of 141 archival paraffin-embedded, formalin-fixed breast tumor tissue samples were obtained from the Clinical Diagnostic Pathology Center of Chongqing Medical University (Chongqing, China). All patients had undergone surgery at the First Affiliated Hospital of Chongqing Medical University during the period ranging from January 2009 to December 2010. The samples of patients treated with neoadjuvant chemotherapy were excluded. Diagnosis was confirmed by immunohistochemical assays for ERα, progestin receptor, and human EGFR2 by the same center. The investigation was approved by the ethics committee of Chongqing Medical University.

### Immunohistochemistry

Commercial rabbit anti-GPER polyclonal antibody (Abcam, Cambridge, MA, USA) was used in immunohistochemical staining as described previously (Filardo *et al*. 2006). Briefly, tissue samples were deparaffinized and heated at 95 °C in 0.1 mol/l sodium citrate (pH 6.0) for antigen recovery, and endogenous peroxidase activity was quenched with 3% H_2_O_2_. Nonspecific binding sites were blocked by incubation in 5% goat serum solution for 30 min at 37 °C. Slides were exposed to primary antibody (1:200) for 2 h at 37 °C. Sections were incubated in HRP-conjugated goat anti-rabbit IgG for 20 min at 37 °C. Diaminobenzidine was added as a substrate. Nuclei were counterstained using Mayer's modified hematoxylin.

GPER expression in stromal fibroblasts and epithelium was evaluated separately. A specimen was considered to be GPER-positive when distinct staining of at least 10% of the stromal fibroblasts was observed (Hoshino *et al*. 2011). GPER expression in tumor cells was evaluated as described previously (Filardo *et al*. 2006).

### Isolation, identification, and immortality of mammary fibroblasts and cell culture

Fibroblasts were isolated as described previously (Zhao *et al*. 2012). Briefly, tumor tissue samples and coupled grossly normal breast tissue samples (at least 2 cm from the tumor margin) resected during surgery were washed with sterile PBS containing antibiotics (100 U/ml penicillin, 100 μg/ml streptomycin, and 50 μg/ml gentamicin) and were then minced with scissors in a culture dish. After digestion with 0.1% collagenase type I (Sigma) at 37 °C for 8–12 h, the suspensions were carefully pipetted up and down in the culture medium. The suspensions were centrifuged (1200 ***g*** for 5 min) and washed with DMEM to remove the fat and tissue debris. The suspension was maintained in DMEM containing 10% fetal bovine serum (FBS) for ∼2 days to allow cell attachment. The most adherent cells (fibroblasts) were further maintained in DMEM containing 10% FBS. The investigation was approved by the Local Ethics Committee, and informed consent was obtained from the patients.

CAFs were identified by immunostaining for α-SMA and fibroblast active protein (FAP), as well as by evaluation of cell morphology (Zhao *et al*. 2012). Fibroblast populations with CAF purity exceeding 85% were used as CAFs in related experiments. The primary CAFs were then immortalized with the human telomerase catalytic subunit (hTERT; from AddGene, Cambridge, MA, USA) for subsequent experiments. MCF-7 cells were maintained in DMEM with phenol red, supplemented with 10% FBS.

### Immunofluorescence

Immunofluorescent staining was utilized to identify CAFs and ER expression following a previously described protocol (Zhao *et al*. 2012). Briefly, 10^5^ cells were grown on coverslips for 24 h and fixed with 4% paraformaldehyde, treated with 0.1% Triton X-100, and blocked with 5% goat serum. The cells were then incubated overnight at 4 °C with primary antibodies targeting α-SMA, FAP, and GPER (Abcam) and ERα and ERβ (ZSGB-BIO, Beijing, China); all dilutions were 1:150. After washing with PBS, the cells were stained with a FITC-labeled goat anti-rabbit secondary antibody (1:100; Zhongshan Golden Bridge, Beijing, China) for 10 min and 4′,6-diamidino-2-phenylindole (DAPI) for 5 min. Immunofluorescent images were obtained using a Nikon Eclipse 80i microscope (Tokyo, Japan).

### Measurement of intracellular Ca^2+^ mobilization

Changes in intracellular Ca^2+^ mobilization were measured using the Ca^2^^+^-sensitive fluorescent probe Fluo-3/AM (1-[2-Amino-5-(2,7-dichloro-6-hydroxy-3-oxo-9-xanthenyl)phenoxy]-2-(2-amino-5-methylphenoxy)ethane-*N,N,N′,N′*-tetraacetic acid, pentaacetoxymethyl ester) (Beyotime, Haimen, Jiangsu, China). Cells were seeded in 35 mm dishes for attachment in normal medium for 24 h. For labeling intracellular Ca^2+^, the cells were incubated in 500 μl DMEM with 2.5 M Fluo-3/AM at 37 °C in the dark for 1 h. The cells were then washed and stored in phenol-free DMEM without Fluo-3/AM for 30 min at 37 °C. Fluorescence (excitation 488 nm and emission 543 nm) was determined using a Leica TCS SP2-laser scanning spectral confocal microscope (Mannheim, Germany). Scanning was performed every 3 s and baseline fluorescence was recorded for 15 s. The test compounds diluted in assay buffer were then added, and fluorescence intensity was monitored for 150 s. Data were quantified and analyzed using Image J Software (National Institutes of Health, Bethesda, MD, USA). Background fluorescence was subtracted, and fluorescence intensity is expressed relative to baseline values.

### Dynamic monitoring of GPCR activation

The cellular response to GPCR stimulation was monitored using an RTCA DP instrument (xCELLigence system, Roche; Leonard *et al*. 2013). The RTCA DP Station was maintained in a humidified cell-culture incubator under normal culture conditions throughout the experiments. Background impedance was measured with 100 μl of cell-culture medium/well. CAFs (2.0×10^4^/well) were plated, and the final volume of the cell-culture medium was adjusted to 200 μl/well. To allow equal distribution of cells, E-Plates 16 containing cells were pre-incubated for 30 min at room temperature. Subsequently, the plates were transferred to the RTCA DP Station inside the incubator and cultured overnight. Impedance was routinely recorded at 15-min intervals to monitor cell-culture conditions. After the administration of GPCR agonists, impedance was monitored at intervals of 1–3 min for at least 2 h. Results are expressed as cell index normalized to the time point of compound administration.

### siRNA transfection

CAFs (4×10^5^) were seeded into 25 cm^2^ culture flasks in 2 ml of growth medium and grown to 80% confluence before transfection. GPER-specific siRNA (siGPER) or nonspecific control siRNA (scrambled siRNA) (Genechem, Shanghai, China) were transiently transfected using Lipofectamine 2000 reagent following the manufacturer's instructions. The target sequences for *GPR30* siRNA were 5′-GCUGUACAUUGAGCAGAAATT-3′ (A) and 5′-UUUCUGCUCAAUGUACAGCTT-3′ (B). The control siRNA sequence that did not match any known human cDNA was 5′-AAGGTGTCAGAAACTGACGAT-3′. GPER protein expression was analyzed by western blotting after transfection.

### Real-time RT-PCR

Total RNA was isolated from cells using TRIzol (Invitrogen) according to the manufacturer's protocol. RNA was reverse-transcribed using the RT2 First Strand Kit and MMLV-RT (Takara, Dalian, China). The cDNA was subjected to real-time PCR amplification using gene-specific primers and 2× Brilliant II SYBR Green QPCR Master Mix (Invitrogen, La Jolla, CA, USA) as described previously (Zhao *et al*. 2012). Primer sequences are listed in Table 1. Specific gene expression was calculated using the comparative 2^−ΔΔ^^*C*^^t^ method with *GAPDH* as the calibrator.

### Proliferation assay

Cells were seeded into 96-well plates (5×10^3^ cells/well) and cultured for 24 h in normal growth medium. The medium was then replaced with phenol red-free and serum-free medium (DMEM; Gibco), and the cells were cultured for a further 24 h before the addition of E_2_, G1, or TAM with or without G15, AG, U0126, and WM pretreatment at the designated concentration. The cells were further cultured in phenol red-free DMEM containing 2.5% dextran-coated charcoal-treated FBS. Cell viability was evaluated using Cell Counting Kit-8 (Beyotime) after 72 h.

### Cell-cycle assay

Cells were seeded in six-well plates (2×10^5^ cells/well) and cultured for 24 h. Cell growth was synchronized in phenol red-free and serum-free medium for 24 h before the addition of E_2_, G1, or TAM with or without G15, AG, U0126, and WM pretreatment at the designated concentration for 24 h. Cell-cycle distribution was analyzed by flow cytometry as described previously (Liu *et al*. 2010). Briefly, treated cells were harvested and fixed with 70% ethanol at 4 °C for 1 h and then resuspended in 1 ml PBS containing propidium iodide (PI; 50 μg/ml) and RNase A (0.1 mg/ml) and incubated at 37 °C for 30 min. Finally, the cells (10^5^ cells/analysis) were analyzed by flow cytometry (FACSVantage SE, BD, Franklin Lakes, NJ, USA), and cell-cycle distribution was determined by PI staining of DNA content.

### Immunoblotting

Cells were stimulated with E_2_, G1, or TAM for 15 min with or without G15, AG, U0126, and WM pretreatment. Western blotting was then performed as described previously (Liu *et al*. 2010). Briefly, cell lysates were harvested in a cell lysis buffer (Boster, Wuhan, China), dissolved in 9% SDS–PAGE buffer, and subjected to western blotting using primary detection antibodies against total or phosphorylated ERK1/2 (diluted 1:1000; BioWorld, St Louis Park, MN, USA) and GPER. Membranes were incubated overnight at 4 °C before incubation with the appropriate HRP-conjugated secondary antibodies. Immunodetection was conducted using the enhanced chemiluminescence system (Amersham Pharmacia Biotech). Optimal density was analyzed using Image J Software, and results were expressed as fold change relative to the control.

### E_2_ production assay

Cells were seeded in six-well plates (2×10^6^ cells/well) and cultured to 50% confluence. Testosterone was added at the appropriate concentration. The administration of G1 or TAM was done 1 h later with or without G15, AG, U0126, and WM pretreatment. The cells were cultured for 48 h, and the medium was harvested for E_2_ detection using the Access E_2_ Immunoassay System (Beckman Coulter, Brea, CA, USA) in the Endocrinology Laboratory, the First Affiliated Hospital of Chongqing Medical University (Chongqing, China).

### Statistical analysis

Statistical analysis was carried out using the SPSS System 17.0 for Windows. Associations between GPER expression and clinicopathological determinants were evaluated using the *χ*^2^ test and the Fisher's exact test (for nominal variables) as appropriate. For measurement data, Student's *t*-test or ANOVAs followed by the Student–Newman–Keuls multiple comparison tests were used to evaluate differences between the subgroups. Two-tailed *P* values ≤0.05 were considered to be statistically significant.

## Results

### GPER is expressed in stromal fibroblasts of primary breast cancer tissues

**Figure 1:**
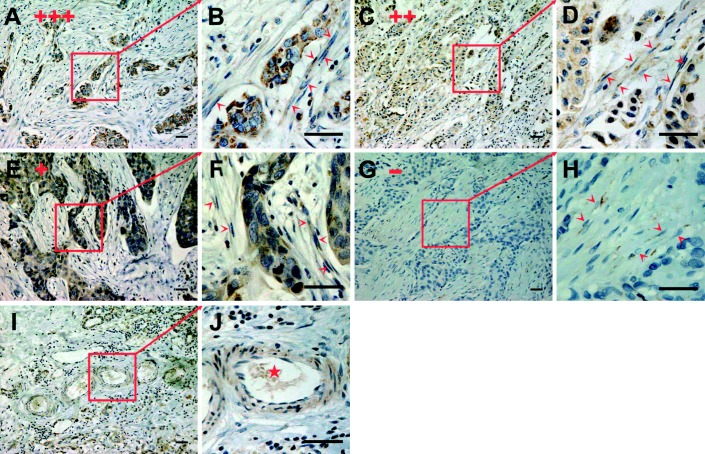
Representative cases of archival paraffin-embedded breast tumor tissue samples immunostained with GPER. GPER was positively stained in stromal fibroblasts (arrows indicate fibroblasts stained by GPER in (B), (D), (F) and (H)) with varying staining density in epithelium. According to the intensity and percentage of positive-staining cells, the expression of GPER in epithelium cells was ranked as one of four grades, +++ (A), ++ (C), + (E), - (G). Immunopositivity was detected in arterial endothelial cells and smooth muscle cells (I, star in J). Scale bars: 50 μm.

All the 141 tumor samples included in this study were invasive ductal carcinoma samples from patients who had not received neoadjuvant chemotherapy. The characteristics of patients and tumors are summarized in Table 2. Stromal fibroblasts were identified as large spindle-shaped mesenchymal cells with stress fibers and well-developed fibronexus based on previously reported descriptions (Hoshino *et al*. 2011). Antibody specificity was validated in SKBr3, MDA-MB-468, MCF-7, MDA-MB-435, and MDA-MB-231 breast cancer cells, which are known to express GPER at varying levels (Supplementary Fig. 1, see section on supplementary data given at the end of this article). GPER was positively stained in a cytoplasmic pattern, and 59 tumor samples (41.8%) were considered to be stromal fibroblast GPER-positive based on distinct staining of 10% or more of the fibroblasts (Table 2). Two-thirds (66.7%) of the samples exhibited positive tumor cell staining of varying density. Varying degrees of epithelial GPER expression were observed (Fig. 1A, B, C, D, E, F, G and H). However, a significant association was detected between stromal and epithelial GPER expression (data not shown). Interestingly, positive GPER staining was also observed in arterial smooth muscle cells and endothelial cells (Fig. 1I and J), which are considered to be the origins of CAFs (Zeisberg *et al*. 2007).

Correlations between GPER expression in stromal fibroblasts, as well as in carcinoma cells, and clinicopathological parameters of breast cancer are summarized in Table 2. GPER staining in stromal fibroblasts did not correlate with the determinants analyzed. However, epithelial GPER expression was exclusively associated with ERα expression.

### GPER is expressed in CAFs isolated from primary breast cancer tissues

**Figure 2:**
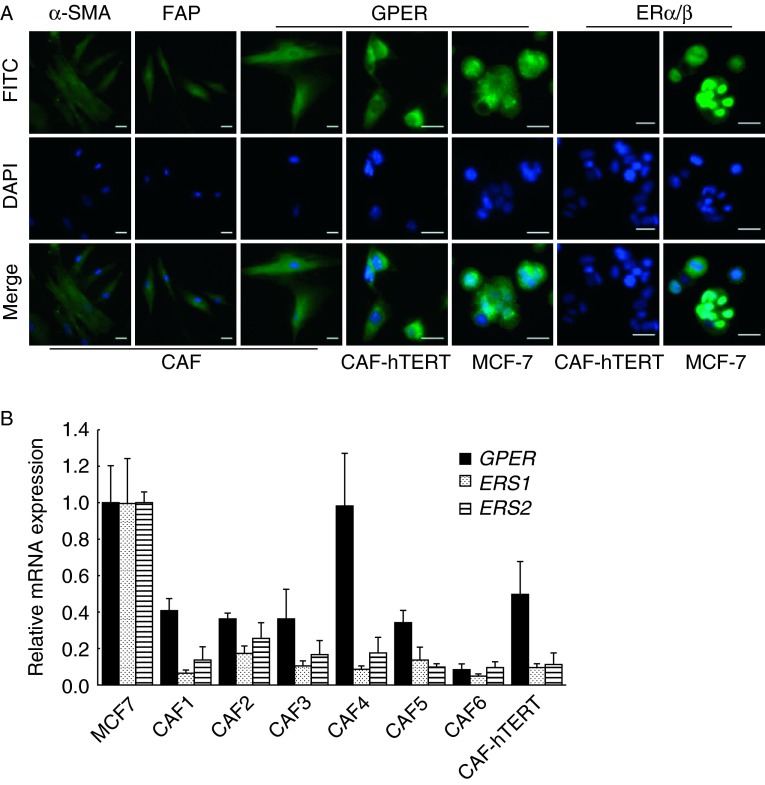
GPER is expressed in primary and immortalized breast CAFs. (A) CAFs were identified by immunofluorescent staining with α-SMA and FAP. ERs were detected in primary breast CAFs, immortalized CAFs (CAF-hTERT), and MCF-7 cells as positive controls. Scale bars: 25 μm. (B) *ER* mRNA expression was evaluated by quantitative real-time RT-PCR in primary CAFs, CAF-hTERT cells, and MCF-7 cells. Gene expression was normalized to *GAPDH*, and results are shown as fold changes of mRNA levels compared with MCF-7 cells. The data are shown as means±s.d. for three independent experiments.

Fibroblasts isolated from primary breast cancer tissues were observed to be myofibroblasts with both a spindle-shaped and a stellate morphology. Mammary CAFs were identified by positive staining for α-SMA and FAP as described previously (Zhao *et al*. 2012; Fig. 2A).

GPER expression was confirmed by immunostaining of primary and immortalized (CAF-hTERT) breast CAFs, using MCF-7 cells as positive controls (Fig. 2A). Moreover, we detected *GPER* mRNA by real-time RT-PCR in six cases of primary CAFs and immortalized CAFs (Fig. 2B). The level of GPER expression in primary CAFs ranged from 0.08- to 0.98-fold (mean, 0.42-fold) relative to the expression detected in MCF-7 cells. However, GPER expression in CAF-hTERT cells was half (0.49-fold) of that detected in MCF-7 cells. Considering that GPER was identified as a GPCR and overexpressed in MCF-7 cells (Carmeci *et al*. 1997), GPER expression in breast CAFs was abundant. Notably, the other two ERs, ERα and ERβ, were not detected by immunostaining or quantitative RT-PCR (Fig. 2A and B), consistent with a previous report (Madeo & Maggiolini 2010).

### E_2_, G1, and TAM stimulate GPER response in breast CAFs

**Figure 3:**
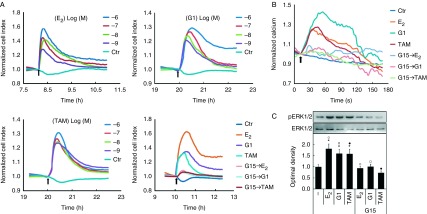
GPER is activated by E_2_, G1, and TAM in breast CAFs. (A) Cell index was monitored dynamically using the xCELLigence system following the administration of E_2_, G1, and TAM (concentrations as indicated or 1 μM for all the three agents) at the indicated time (arrow) with or without pretreatment with G15 (1 μM). (B) Ca^2+^ labeled with the Fluo-3/AM probe was monitored dynamically by laser scanning spectral confocal microscopy. E_2_ (10 nM), G1 (1 μM), and TAM (10 nM) were added at the indicated time (arrow) with or without G15 (1 μM) pretreatment. (C) CAFs were treated with E_2_ (10 nM), G1 (1 μM), and TAM (10 nM) for 15 min, with or without G15 (1 μM) pretreatment. Cell extracts were used for immunoblotting analysis of total and phosphorylated ERK1/2. Results are shown as fold changes in optimal density compared with the control (−). *P<0.05, vs control; open square, open cirlce, and filled circle, *P*<0.05, between the two marked groups.

E_2_ and TAM have been recognized as agonists of GPER, while G1 has been identified as a selective agonist (Wang *et al*. 2010, Prossnitz & Barton 2011). The xCELLigence system has been demonstrated to be reliable and sensitive for the analysis of GPCR activation in living cells (Scott & Peters 2010). To confirm the effect of E_2_, G1, and TAM on GPER in breast CAFs, GPCR activation was monitored dynamically using the xCELLigence system following the administration of these agonists. E_2_, G1, and TAM stimulated transient and dose-dependent cell index increases within 1 h, with peaking being observed at 1.58±0.21, 1.29±0.11, and 1.31±0.09 respectively. These responses were eliminated by pretreatment with the GPER-specific antagonist G15 (Fig. 3A).

Ca^2+^ modulation has been reported to be correlated with GPER stimulation (Pietras *et al*. 2005) and therefore has been utilized as a sensor of GPER activation in early studies (Revankar *et al*. 2005). We monitored intracellular Ca^2+^ modulation by labeling laser scanning spectral confocal microscopy analysis of the Fluo-3 AM probe in CAFs. Within 30 s, E_2_ (1 μM), G1 (1 μM), and TAM (1 μM) enhanced fluorescence intensity (fold changes: 1.41±0.12, 1.29±0.08, and 1.26±0.09, respectively, relative to the baseline), indicating that these agonists stimulated intracellular Ca^2+^ modulation. Similarly, G15 pretreatment blocked the effects on intercellular Ca^2+^ stimulated by E_2_, G1, and TAM (Fig. 3B).

It has been demonstrated that GPER induces ERK1/2 phosphorylation in response to E_2_ in breast cancer cells (Filardo *et al*. 2000). We also observed that E_2_ (1 μM), G1 (1 μM), and TAM (1 μM) stimulated ERK1/2 phosphorylation (fold changes: 1.82±0.22, 1.60±0.19, and 1.59±0.21, respectively, relative to the control). Moreover, G15 pretreatment inhibited ERK1/2 activation induced by GPER ligands in breast CAFs (Fig. 3C).

Although GPER was also detected in fibroblasts isolated from normal breast tissues, no significant effects of E_2_, G1, and TAM were observed in the aforementioned assays (Supplementary Figs 2 and 3, see section on supplementary data given at the end of this article).

### E_2_, G1, and TAM promote GPER-mediated proliferation in breast CAFs

**Figure 4:**
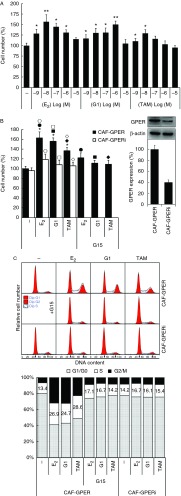
The EGFR/ERK axis is involved in GPER-mediated proliferation in breast CAFs. (A and B) Synchronized cells were cultured with E_2_ (concentrations as indicated or 10 nM), G1 (concentrations as indicated or 1 μM), or TAM (concentrations as indicated or 10 nM) for 72 h, with or without G15 pretreatment (1 μM). Cell viability was then measured using Cell Counting Kit-8. *P<0.05 vs control; **P<0.01 vs control; filled circles, filled squares, filled diamonds, open circles, open squares, and open diamonds, *P*<0.05, between the two marked groups. (C) Synchronized cells were cultured with E_2_ (10 nM), G1 (1 μM), and TAM (10 nM) in the presence or absence of G15 (1 μM) for 24 h. The cells were then stained with propidium iodide and cell-cycle distribution was analyzed by flow cytometry. A full colour version of this figure is available via http://dx.doi.org/10.1530/ERC-13-0237.

The role of GPER in CAF proliferation was examined by the administration of E_2_, G1, and TAM, and cell viability was measured after treatment for 3 days. E_2_, G1, and TAM enhanced the proliferation of breast CAFs after culture for 72 h in a dose-dependent manner. Maximal proliferation of 162.7±12.1, 155.8±6.9, and 136.6±8.5% relative to the control was detected following treatment with E_2_ (10 nM), G1 (1 μM), and TAM (10 nM) respectively (Fig. 4A). G15 abolished these cell proliferative effects (Fig. 4B). To confirm the role of GPER in these proliferative effects further, *GPER* was knocked down to 39% by specific siRNA transfection of CAFs (CAF-GPERi). Interestingly, none of the ligands stimulated significant proliferative effects in CAF-GPERi cells. Moreover, CAF-GPERi cell numbers were significantly less than CAF-GPER cell numbers following the administration of E_2_ (10 nM), G1 (1 μM), and TAM (10 nM) (Fig. 4B).

The GPER-mediated effect on cell-cycle progression was investigated. Mammary CAFs were synchronized by estrogen and serum withdrawal and then treated with E_2_ (10 nM), G1 (1 μM), and TAM (10 nM) for 24 h followed by PI staining and flow cytometry. The administration of E_2_, G1, and TAM significantly increased the proportion of S-phase CAFs from 13.4±3.2% to 26.9±6.8, 24.7±5.4, and 28.6±4.6%. Importantly, both G15 pretreatment and *GPER* knockdown blocked the accumulation induced by E_2_, G1, and TAM (Fig. 4C).

### GPER/EGFR/ERK pathway is involved in TAM-induced CAF proliferation

**Figure 5:**
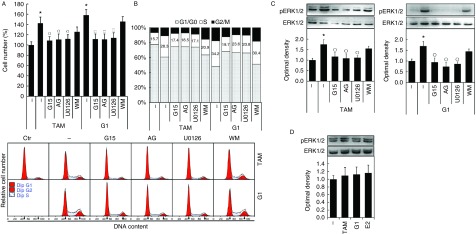
The EGFR/ERK axis is involved in GPER-mediated proliferation in breast CAFs. (A) Synchronized cells were cultured with G1 (1 μM) or TAM (10 nM) in the presence or absence of G15 (1 μM), AG (10 μM), U0126 (10 μM), and WM (10 μM) for 72 h. Cell viability was measured using Cell Counting Kit-8. *P<0.05 vs control; open circles, *P*<0.05 vs TAM group; and open squares, *P*<0.05 vs G1 group. (B) Synchronized cells were cultured with G1 (1 μM) or TAM (10 nM) in the presence or absence of G15 (1 μM), AG (10 μM), U0126 (10 μM), and WM (10 μM) for 24 h. The cells were then stained with propidium iodide and cell-cycle distribution was analyzed by flow cytometry. (C) The cells (CAF-GPER) were treated with G1 (1 μM) or TAM (10 nM) for 15 min, with or without G15 pretreatment (1 μM), AG (10 μM), U0126 (10 μM), and WM (10 μM). Cell extracts were used for immunoblotting analysis of phosphorylated and total ERK1/2. Results are shown as fold changes in optimal density compared with the control. *P<0.05 vs control and open circles, *P*<0.05 vs G1 and TAM group. (D) The cells (CAF-GPERi) were treated with E_2_ (10 nM), G1 (1 μM), and TAM (10 nM) for 15 min. Cell extracts were used for immunoblotting analysis of phosphorylated and total ERK1/2. Results are shown as fold changes in optimal density compared with the control. A full colour version of this figure is available via http://dx.doi.org/10.1530/ERC-13-0237.

As has been mentioned previously, GPER stimulation is involved in the transactivation of EGFR/ERK signaling and PI3K modulation in breast cancer cells (Filardo *et al*. 2002, Revankar *et al*. 2005). In this study, G15 (selective GPER antagonist), AG, U0126, and WM (inhibitors of EGFR, ERK1/2, and PI3K respectively) were used to evaluate the role of these pathways in GPER-mediated proliferation and cell-cycle changes in breast CAFs. G15, AG, and U0126 significantly inhibited the proliferation induced in CAFs by G1, TAM (Fig. 5A), and E_2_ (data not shown). WM treatment had no significant influence on CAF proliferation (Fig. 5A). Similar trends were observed for the accumulation of S-phase cells in response to G1, TAM (Fig. 5B), and E_2_ (data not shown). As a sensor of GPER/EGFR/ERK signaling activation, ERK1/2 phosphorylation was detected by immunoblotting. As expected, G15, AG, and U0126 significantly reduced ERK1/2 phosphorylation induced by G1, TAM (Fig. 5C), and E_2_ (data not shown) in CAFs. However, WM treatment had no significant influence on ERK1/2 phosphorylation in CAFs (Fig. 5C). Moreover, these GPER ligands did not enhance ERK phosphorylation in CAF-GPERi cells (Fig. 5D).

### GPER is involved in TAM-induced E_2_ production

**Figure 6:**
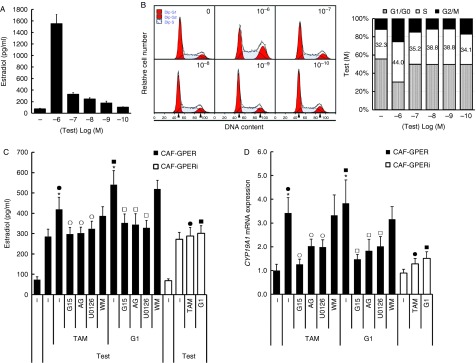
Effect of GPER/EGFR/ERK signaling on estradiol (E_2_) production in breast CAFs. (A and B) Testosterone was added to CAF culture medium for 48 h before harvesting for E_2_ detection by chemiluminescence immunoassay. Cell-cycle distribution was analyzed by flow cytometry. (C) Testosterone (100 nM) was added to breast CAF culture medium and cells were cultured with G1 (1 μM) or TAM (10 nM) in the presence or absence of G15 (1 μM), AG (10 μM), U0126 (10 μM), and WM (10 μM) for 48 h. The medium was harvested for E_2_ detection by chemiluminescence immunoassay. *P<0.05 vs cells treated with only testosterone; open circles, *P*<0.05 vs TAM group; open squares, *P*<0.05 vs G1 group; and filled circles and filled squares, *P*<0.05, between the two marked groups. (D) Synchronized cells were cultured with G1 (1 μM) or TAM (10 nM) in the presence or absence of G15 (1 μM), AG (10 μM), U0126 (10 μM), and WM (10 μM) for 18 h. Total RNA was then extracted to perform quantitative RT-PCR analysis of *CYP19A1* mRNA expression. Gene expression was normalized to *GAPDH*, and results are shown as fold changes of mRNA levels compared with the control. *P<0.05 vs control; open circles, *P*<0.05 vs TAM group; open squares, *P*<0.05 vs G1 group; and filled circles and filled squares, *P*<0.05, between the two marked groups. A full colour version of this figure is available via http://dx.doi.org/10.1530/ERC-13-0237.

CAFs are known to be an important source of local estrogen in breast cancer (Yamaguchi & Hayashi 2009). We tested the ability of CAFs to synthesize E_2_ *in vitro*. The substrate of E_2_, testosterone, was added to the CAF culture medium for 48 h before harvesting for E_2_ detection by chemiluminescence immunoassay. The administration of testosterone stimulated dose-dependent E_2_ production. Furthermore, at a dose of 10 mM testosterone, the S-phase accumulation increased dramatically to 44±5.8% (vs 32.3±4.5% in the control), while the concentration of E_2_ increased to 1553±158 pg/ml (vs 74±11 in the control) (Fig. 6A).

Increasing evidence indicates that GPER is involved in aromatase gene regulation and local estrogen production (Lin *et al*. 2009, van Duursen *et al*. 2011, Pupo *et al*. 2012). We evaluated the potential role of GPER and related signaling in E_2_ production in the breast microenvironment. Interestingly, GPER activation increased E_2_ production in breast CAFs. Treatment of CAFs with testosterone (100 nM) stimulated the production of 286±36 pg/ml (vs 73±14 pg/ml in the control) of E_2_ after cultivation for 48 h. The administration of TAM (10 nM) and G1 (1 μM) further increased the concentration of E_2_ to 419±60 and 541±69 pg/ml respectively. This enhancement was inhibited by *GPER* knockdown and G15, AG, and U0126 administration but not by WM administration (Fig. 6C). These data were paralleled by the effects of *GPER* interference and G15, AG, U0126, and WM on cell viability when administered under identical conditions (Fig. 5B). Hence, we attempted to verify whether this phenomenon was caused by GPER-mediated proliferation or GPER-induced aromatase expression in breast CAFs (Lin *et al*. 2009, van Duursen *et al*. 2011, Pupo *et al*. 2012). As expected, culturing with TAM (10 nM) and G1 (1 μM) for 18 h induced *CYP19A1* gene expression (fold changes: 3.4±0.6 and 3.8±1.0, respectively, vs the control) in CAFs, and this effect was blocked by *GPER* interference and G15, AG, and U0126 but not by WM (Fig. 6D).

## Discussion

Estrogens play an important role in breast cancer development. ERα is widely accepted as a target for endocrine therapy of breast cancer, and a well-known SERM, TAM, provides considerable benefits for patients with breast cancer at different stages. However, acquired TAM resistance in ERα-positive breast cancer cells has become a significant challenge in its clinical application. Recently, TAM resistance has been reported to be correlated with the expression of the novel ER, GPER (Ignatov *et al*. 2010, 2011). In the present study, we demonstrated that GPER is expressed in the stromal fibroblasts of primary breast cancer tissues and CAFs isolated from tumor tissues. TAM, in addition to E_2_ and the GPER agonist G1, promoted proliferation, cell-cycle progression, and E_2_ production via the GPER/EGFR/ERK axis in breast CAFs, providing novel insights into the GPER-mediated CAF-dependent mechanism of TAM resistance in breast cancer.

GPER was first detected as a *GPCR* gene in breast cancer cell lines as well as in primary breast cancer in 1997 (Carmeci *et al*. 1997). Subsequently, several examples of GPER expression in breast cancer have been reported. Filardo *et al*. (2006) conducted an immunohistochemical analysis of the distribution of GPER in 361 cases of breast cancer and correlated GPER with the expression of ERα, the progestin receptor, and human EGFR2 as well as tumor size and the presence of metastasis. However, results obtained in other studies were not consistent with these observations (Tu *et al*. 2009, Arias-Pulido *et al*. 2010, Ignatov *et al*. 2011). In the present study, the presence of GPER in cancer cells was exclusively correlated with ERα expression (Table 2). Additionally, the co-expression of GPER and ERα in carcinoma cells was found in 41.1% of all samples, which is in accordance with previous reports (Filardo *et al*. 2006, Tu *et al*. 2009, Ignatov *et al*. 2011). The presence of ERα is considered to be a definite indication for the administration of TAM, which is a well-characterized agonist of GPER. TAM administration has been shown to stimulate GPER in these patients, resulting in proliferative effects (Lin *et al*. 2009, Madeo & Maggiolini 2010, Pupo *et al*. 2012, Vivacqua *et al*. 2012). *In vitro*, TAM has been reported to promote proliferation via sensitivity-enhanced GPER/EGFR/MAPK signaling in TAM-resistant MCF-7 cells (Ignatov *et al*. 2010, Peralta *et al*. 2010). In recent follow-up studies, GPER has been shown to be correlated with reduced relapse-free survival in patients undergoing TAM treatment (Ignatov *et al*. 2011). These observations implicate GPER in breast carcinoma TAM resistance, although this remains to be confirmed.

GPER has been detected in stromal inflammatory cells, myoepithelium, and fibroblasts in addition to tumor cells of primary breast cancer (Arias-Pulido *et al*. 2010), although an analysis of its expression has not been reported previously. In this study, positive GPER staining was observed in stromal fibroblasts, smooth muscle cells, and arterial endothelial cells, all of which are recognized as origins of CAFs (Karnoub *et al*. 2007, Xing *et al*. 2010, Cirri & Chiarugi 2012). Considering the important role of both estrogen and CAFs in breast cancer development, we correlated GPER expression in stromal fibroblasts with the clinicopathological determinants of breast cancer. Although no significant association was found, GPER expression was definitely detected in stromal fibroblasts of 41.8% of the samples (Table 2), implying a GPER-mediated CAF-dependent estrogenic effect in tumor microenvironment.

Estrogens play an important role in the development of mammary glands and associated carcinomas. In healthy women, the extracellular matrix of breast tissue, in addition to the mammary epithelial cells, undergoes cyclic changes with each menstrual cycle (Arendt *et al*. 2010). Although stromal ERα has been demonstrated to be critical for breast development in mice (Mueller *et al*. 2002), studies focusing on ERs and estrogen effects in tumor-associated stroma are rare. Recently, GPER has been demonstrated to be a unique ER mediating E_2_-stimulated proliferation and migration of breast CAFs (Madeo & Maggiolini 2010). Subsequently, GPER/EGFR/ERK signaling has been claimed to upregulate the expression of EGR1, CTGF, C-FOS, and cyclin D1, resulting in proliferation enhancement in mammary CAFs (Pupo *et al*. 2012, Vivacqua *et al*. 2012). Interactions between estrogen and growth factors were delineated decades ago (Pietras *et al*. 2005). GPER is thought to be largely responsible for this crosstalk, as it has been demonstrated to employ the EGFR/ERK pathway as its predominant signaling pathway, and growth factors including EGF, CTGF, transforming growth factor α/β, and insulin-like growth factor have been observed to regulate GPER expression or be regulated by GPER activation (Filardo 2002, Filardo *et al*. 2002, Vivacqua *et al*. 2009, Madeo & Maggiolini 2010, De Marco *et al*. 2012). Similarly, we demonstrated in this study that GPER/EGFR/ERK signal transduction mediates E_2_-induced proliferation in breast CAFs, based on the observation that G1 induced similar proliferative and cell-cycle promotion that was blocked not only by *GPER* interference but also by G15, AG, and U0126 administration. These observations revealed the CAF-dependent estrogenic effects on breast cancer microenvironment.

Interestingly, we demonstrated that TAM also stimulated the proliferation of CAFs via the GPER/EGFR/ERK pathway in this study (Fig. 5), and its metabolite 4-hydroxytamoxifen has also been shown to promote breast CAF growth in a previous study (Vivacqua *et al*. 2012). Notably, ∼40% of ERα-positive tumors expressed GPER in stromal fibroblasts in our study (Table 2). CAFs are promoters of tumor growth; therefore, it can be suggested that the anti-estrogen effect of TAM in tumor cells is negated by TAM-induced proliferation in CAFs, resulting in acquired TAM resistance in these patients. Recently, GPER expression in breast cancer cells has been reported to be correlated with poorer relapse-free survival only in patients treated with TAM, but tended to be a favorable factor in patients who did not receive TAM therapy (Ignatov *et al*. 2011). A potential role for GPER expression in CAFs in this bidirectional effects is indicated by the detection of stromal fibroblast GPER expression in 40% of ERα-positive tumors in our study. Thus, more follow-up data are required in addition to an analysis of GPER expression in stromal fibroblasts and epithelium as determinants for TAM administration in breast cancer patients.

CAFs are an important source of local estrogen, which is an important mediator of breast cancer progression (Yamaguchi & Hayashi 2009). As a key enzyme involved in estrogen synthesis, aromatase has become a paradigm target of endocrine therapy in breast cancer patients. Intriguingly, GPER has been reported to be correlated with aromatase gene upregulation directly or indirectly in previous studies (van Duursen *et al*. 2011, Pupo *et al*. 2012). An analog and a metabolite of TAM have been demonstrated to promote aromatase gene expression via GPER activation in endometrial cancer cells (Lin *et al*. 2009). Furthermore, genistein, a well-recognized GPER agonist, has been reported to increase breast-cancer-associated aromatase expression and activity *in vitro* (van Duursen *et al*. 2011). In the present study, TAM and G1 increased both *CYP19A1* mRNA and E_2_ production in CAFs, and this effect was dependent on GPER/EGFR/ERK pathway signaling. Taken together, these observations indicate that GPER in breast CAFs is involved in the increased local production of E_2_, thus presenting a feed-forward loop model that has been postulated to contribute to TAM resistance in breast cancer patients.

The role of GPER in breast CAFs warrants further investigation following its detection in stromal fibroblasts in breast cancer tissues. Furthermore, we confirmed previous reports that the GPER/EGFR/ERK pathway contributes to the proliferation of breast CAFs (Madeo & Maggiolini 2010, Pupo *et al*. 2012, Vivacqua *et al*. 2012). Thus, our data provide new insights into estrogenic effects on tumor microenvironment. More importantly, TAM, which is commonly accepted to be a SERM for breast cancer patients, was shown to stimulate proliferation and E_2_ production in breast CAFs. Hence, GPER is implicated in TAM resistance in a CAF-dependent manner. As such, GPER expression in stromal fibroblasts would be considered a contra-indicator of TAM application or additional therapy targeting GPER signaling ought to be offered in the future.

## Supplementary data

This is linked to the online version of the paper at http://dx.doi.org/10.1530/ERC-13-0237.

## Figures and Tables

**Table 1:** Real-time PCR primer sequences for genes analyzed

**Genes**	**Forward**	**Reverse**
*GPER*	TGGGGAAGAGGCCACCA	CGT GGAGCTGCTCACTCTCTG
*ERα*	AGGCCAAATTCAGATAATCGAC	GAAGCATAGTCATTGCACAC
*ERβ* (*ESR2*)	AGCTCAGCCTGTTCGACCAAG	ACGCATTTCCCCTCATCCCT
*CYP19A1*	GGGCACATCCTCAATACCAG	CAGAAGGGTCAACACGTCCA
*GAPDH*	GAAGGTGAAGGTCGGAGTC	GAAGATGGTGATGGGATTTC

**Table 2:** Patient and tumor characteristics

	**No. of patients** (%)	**GPER positivity**	
Parenchymal	Stromal
Total	141	94	59
Age (mean±s.d.)	53.0±10.9	53.6±10.9	53.3±11.8
Menstruation status			
Estrous	52 (36.9)	34	20
Postmenopause	89 (63.1)	60	39
Tumor size	25.6±13.1	24.4±10.9	26.4±14.8
<2 cm	58 (41.1)	42	22
2–5 cm	75 (53.2)	47	32
>5 cm	8 (5.7)	5	5
pN^(^^*n*^^=^^134)^			
0	72 (51.1)	50	29
1	28 (19.9)	19	13
2	22 (15.6)	12	11
3	12 (8.5)	8	5
Staging			
I	35 (24.8)	25	14
II	61 (43.3)	42	25
III	38 (27.0)	22	19
X	7 (5.0)	5	1
Histological grade			
1	25 (17.7)	15	12
2	94 (66.7)	64	36
3	22 (15.6)	15	11
NPI (*n*=134)	4.2±1.2	4.1±1.2	4.3±1.3
1	13 (9.2)	6	7
2	36 (26.9)	29	13
3	63 (44.7)	41	26
4	22 (15.6)	13	12
ER			
+	79 (56.0)	58*	32
−	62 (44.0)	36	27
PR			
+	58 (41.1)	40	24
−	83 (58.9)	54	35
HER2			
−	67 (47.5)	45	29
+	20 (14.2)	13	10
++	31 (22.0)	24	13
+++	23 (16.3)	12	7

*P=0.041. NPI, Nottingham prognostic index.
